# Analysis of Wear Resistance of Borided Steel C45

**DOI:** 10.3390/ma13235529

**Published:** 2020-12-03

**Authors:** Mykhaylo Pashechko, Krzysztof Dziedzic, Jerzy Jozwik

**Affiliations:** 1 Department of Fundamental of Technology, Fundamentals of Technology Faculty, Lublin University of Technology, 20-618 Lublin, Poland; m.pashechko@pollub.pl; 2 Department of Computer Science, Electrical Engineering and Computer Science Faculty, Lublin University of Technology, 20-618 Lublin, Poland; 3 Department of Production Engineering, Mechanical Engineering Faculty, Lublin University of Technology, 20-618 Lublin, Poland; j.jozwik@pollub.pl

**Keywords:** borided coating, wear, segregation, wear mechanism

## Abstract

The wear resistance of diffusion coatings in conditions of specific pressures of 3, 7 and 10 MPa was studied. The boride coatings were prepared by means of diffusion methods using C45 steel as the substrate material. Research on the microstructure and redistribution of chemical elements on wear surface of a borided layer was carried out. It was found that the boride coatings should be used under a specific pressure of 7 MPa. It was found that the wear of friction couple coating of steel C45 under specific pressure of 3 MPa proceeds according to the oxidation wear mechanism, while under specific pressures of 7 and 10 MPa the abrasive wear prevails. The wear-induced segregation of atoms in coatings was studied using secondary mass-spectroscopy method (SIMS). Increased C, O, and B concentrations were noticed at the wear surface on depth from 50 to 2000 Å. The secondary wear-induced structure formation on the wear surface resulted in high wear resistance of diffusion borided coatings.

## 1. Introduction

Sliding wear is a critical mechanism of material wear loss that occurs in any engineering applications. It is a serious problem for the industry [1,2,3,4,5]. Fe-based hard-facing alloys are widely used to protect machine components exposed to various loading conditions [6,7,8]. Elements of machines and mechanisms operating under significant mechanical loading must have sufficient service life, which is limited mainly by their wear resistance. In order to improve the durability of friction units, it is of scientific and practical interest to study the wear process and wear mechanisms of composite materials, alloys and coatings based on iron, nickel, cobalt and aluminum and which contain carbon, boron, silicon and other elements [9,10,11,12,13,14,15].

Among the methods to increase wear resistance of machine elements is boriding [16]. Borided layers are produced mainly on steel by thermo-chemical processing or by diffusion of reactive boron into the material. Diffusion boriding is the saturation of the surface layer with boron. The process temperature is 900–1000 °C, and its duration is usually several hours. Liquid or solid media that contain and may release boron are most often used for boriding [17,18]. In [17], for AISI-1045 steel boriding, 70% Borax (Na_2_B_4_O_7_) + 30% B_4_C was used as a liquid agent. On the other hand, 5% B_4_C + 90% SiC + 5% KBF_4_ was used for solid-phase boriding. In this way, a boron enriched surface layer is obtained, its thickness varies from 0.03 to 0.15 mm [17,19]. Boron concentration is an important feature during the boronizing process. It affects the formation of Fe_2_B and FeB diffusion layer [20,21]. In the case of FeB-containing layer formation, its hardness can be even 2000–2400 HV. In the case of the layer containing Fe_2_B borides, its hardness ranges from 1600 to 2000 HV [22,23]. High hardness and lower friction coefficient of borided steel makes it a good material for tribological applications. An important drawback of the borided layer is its brittleness, which may cause surface flaking and chipping; for this reason, wear rate may increase.

The methods of preventing brittleness include the production of a single-phase layer composed of Fe_2_B, adjusting the carbon content under the iron boride layer or laser treatment of the borided layer [24]. The carbon content under the iron borides depends on steel chemical composition and may be increased by carburizing prior to boronizing (carboboriding). Laser boronizing is a method where the surface layer of the material is melted together with the alloying material, which is most often preplaced to the surface as a paste mixture containing amorphous boron [25]. The great advantage of this method is the possibility of carrying out the boronizing process locally on the selected surface of machine element [21,26,27]. In [28], Yushkov et al. used electron-beam evaporation of boron-containing targets for boriding. The obtained coating thickness was 3–4 µm.

In [29], Wu et al. studied the tribological properties of Al_0.1_CoCrFeNi high-entropy alloys subjected to boriding. The packboriding method was used. Under dry friction conditions, the authors observed a change in the wear mechanism from abrasive with delamination to the wear mechanism with a visible surface polishing effect. The created boronized layer had a thickness of 50 µm [29,30].

Formation of B_4_C boron carbide in the surface layer is often used to improve tribological properties of various materials [31]. Tang et al. in [32] studied the wear of Al-5083 composite material strengthened by B_4_C boron carbides. Tribological tests were conducted using the pin-on-disc test method, at a load of 3.98–6.37 MPa and sliding speed 0.6–1.25 m/s. The authors indicate an increase in wear resistance with the use of 10 wt.% B_4_C instead of 5 wt.%. The study demonstrates that the upper part of the active layer is rich in Fe, O and B. This proves material transfer from the steel disk to the pin surface, as well as oxidation of the worn surface during the wear test. The authors also observed a wear mechanism of the tested composite materials, which includes adhesive, abrasive and fatigue wear. A very low friction coefficient for mating steel surfaces, namely 0.03–0.05, is mentioned by Erdemir et al. [33]. They describe ultralow friction mechanisms of a surface film on B_4_C. A thin film was observed on the surface, which is formed as a result of reactions of B_4_C with oxygen and of B_2_O_3_ with moisture. It has been shown that B_4_C oxidizes at temperatures above 600 °C. Then, a B_2_O_3_ layer is formed. On cooling down to room temperature, boric acid H_3_BO_3_ is formed. The reduction in sliding wear can be significantly influenced by the formation of oxides on the wear surface, as it was shown in the work [34]. This process is influenced by the heat generated in the friction couple contact area and the heat generated during wear process. Under the heat influence, metallic particles may oxidize and consequently change the wear mechanism.

An interesting phenomenon from the point of view of increasing the wear resistance of materials with boron-containing surface layers is the segregation of some particular atoms on the wear surface. Pashechko et al. observed this process for Fe-Mn-C-B eutectic alloy [35]. Si, Cr and Ni were used as alloying elements. Surface segregation of Si, C and also Al was observed [36]. It has been noted that the effect of carbon atom segregation on the wear properties of steel is unclear. On the one hand, the presence of carbon in the friction surface reduces the adhesive interaction of contacting solids. On the other hand, it may reduce the lubrication efficiency [37,38,39,40,41,42].

In this paper, the results of research on wear resistance, structure and elements redistribution with the application of the secondary ion mass spectroscopy of borided coating on steel C45 surface are presented. This method made it possible to obtain surface and in-depth distribution pattern of chemical elements in the borided layer with the resolution of one to several interatomic distances. The aim of this analysis is to prove that during friction of diffusion boride coatings, the segregation of B, C and O atoms occurs similarly to that during the friction of Fe-Mn-C-B-Si-Ni-Cr eutectic alloys [43,44,45]. The obtained surface has a nanocrystalline structure. This phenomenon results in high wear resistance of diffusion borided coatings.

## 2. Materials and Methods

The wear-resistant coatings were obtained by means of boron thermal diffusion (powderpack method) into the steel C45 substrate surface. The chemical composition of this material is presented in Table 1. The chemical composition of steel C45 [46] comes from a certificate issued by the manufacturer. C45 steel is a plain-carbon steel containing no alloying elements. This material has limited weldability (requires special welding procedures, including preheating and post treatment), is easily machined and hot-formed. It is used for the manufacturing of some tools and machine elements that operate under moderate loads. This steel is a wear resistant material.

The specimens for boriding were cut from C45 steel bars and machined to the required size. Prior to boriding, the surface of the samples was mechanically cleaned by sand-paper. Powder-pack boriding was performed in an airtight container. For solid-phase boriding, a powder mixture based on technical boron carbide and borax 84% B_4_C + 16% Na_2_B_4_O_7_ was used. Before thermochemical processing, all the powder components were dried and milled. The containers were air tightened with boron oxide B_2_O_3_, silicon lump and crushed oxides. During heating of the container, the indicated components created a barrier for oxygen intake. The process temperature was 950–1050 °C, and the saturation time t = 6 h. The thickness of the obtained surface layer after boriding was approximately 110 µm. Boriding is particularly to provide surface hardness and wear-resistance of machine elements. High wear resistance is among the basic goals achieved by boriding.

The study of microstructure and chemical composition of wear-induced layers formed on the surface of both eutectic coating and borided steel was performed using a method of Auger spectroscopy with JAMP-10S equipment (JEOL, Peaboy, MA, USA). Samples were cut parallel to wear surface, and cylinders with a height of not more than 10 mm were placed into the chamber. In the case of the counterpart, segment-shaped sections with no one dimension reaching 10 mm were fabricated.

After air evacuation, the retained pressure was 5 × 10^−7^ Pa. The elemental analyses were conducted using pure metals sensitivity factors. The studied area was bombarded by argon ion beam (voltage ~3 kV, ion current 10^−5^ A); after this, the spectra were registered. The areas for spectral analyses were selected based on chemical structure. They were calculated by statistical analyses of measured spectra. The method of secondary electron recording was also employed. A detector forming an image depending on the composition of the examined surface was used in this study.

Mass-spectrometry examinations (SIMS) were performed by INA3 instrument (Leybold-Heraeus, Cologne, Germany). Samples were fractured in a previously evacuated vacuum chamber and then analyzed in the direction from the depth towards the wear surface. The etching rate of samples was 5–10 Å/s with a target voltage of 200–300 V. The energy of the beam was 3–5 keV. The current of plasma was 3 × 10^−3^ mbar at a pressure of 10^−5^ mbar; etching spot diameter was 3–5 mm.

**Figure 1:**
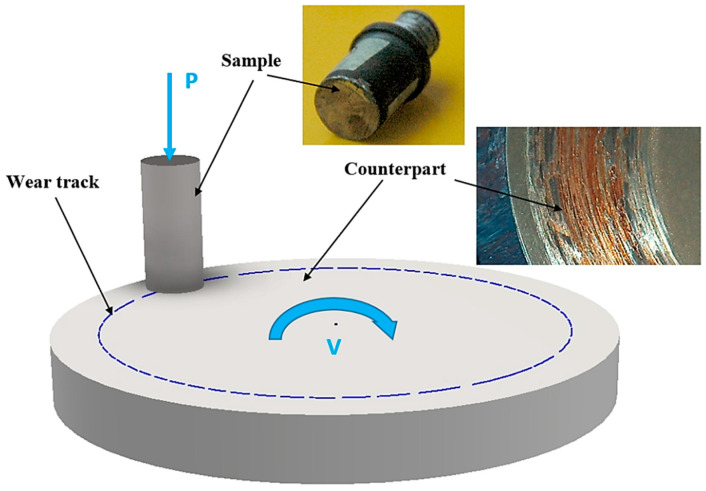
Illustration of a tribological pin on disc test.

A wear test was conducted using pin-on-disk test lay-out. The pin (specimen) diameter was 10 mm; the disk (counterpart) diameter was 50 mm. The contact ratio during the test was 0.2. Both pins and the counterpart were manufactured of steel C45. Pins were subjected to the boriding procedure described above, while counterpart disks were water quenched and tempered at 200 °C; the hardness value was 52–54 HRC (Hardness Rockwell C). The contact pressure used for the test was 3, 7 and 10 MPa; the sliding speed was 0.6 m/s; the time of the wear test was 6 h. For each value of load, we performed 10 separate wear tests. The test apparatus layout, pin specimen and disk counterpart are shown on Figure 1.

## 3. Results and Discussion

**Figure 2:**
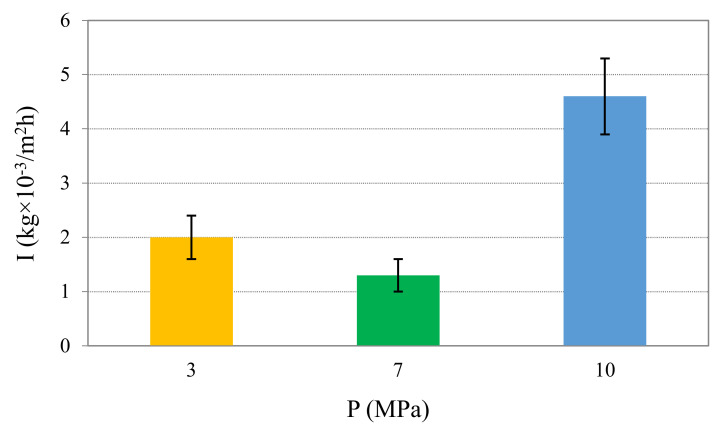
Wear intensity of borided diffusion layer on steel C45.

The wear intensity of borided coating is the lowest during testing at a specific pressure of 7 MPa (1.3 kg × 10^−3^/m^2^h). The wear intensity after wear testing at 3 MPa was 2 kg × 10^-3^/m^2^h. The highest value of wear intensity was during tests at 10 MPa: 4.6 kg × 10^−3^/m^2^h (Figure 2).

The structure and phase composition and elements distribution on the wear surface were studied by means of secondary neutral mass spectroscopy (INA3), microstructural, micro X-ray spectral (Superprobe-733) (JEOL, Peaboy, MA, USA) and micro X-ray phase (DRON-3M) analyses (Bourevestnik JSC, Saint-Petersburg, Russia). This made it possible to analyze the processes developing on contact surfaces during friction of borided coatings.

**Figure 3:**
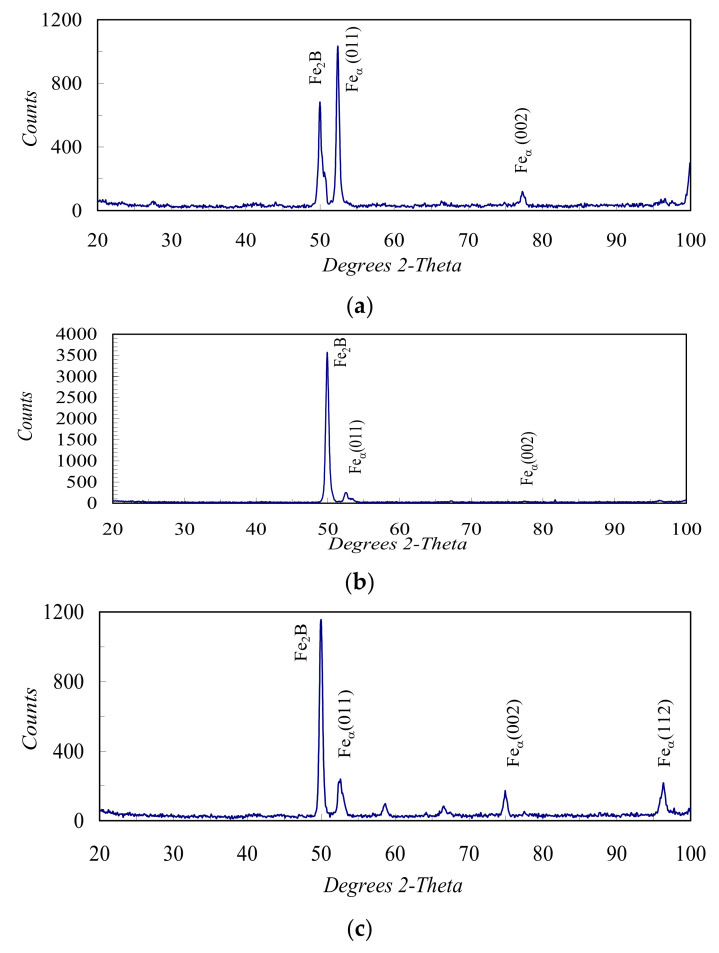
Diffraction patterns of coatings after wear tests at specific pressure: (**a**) 3, (**b**) 7 and (**c**) 10 MPa.

**Figure 4:**
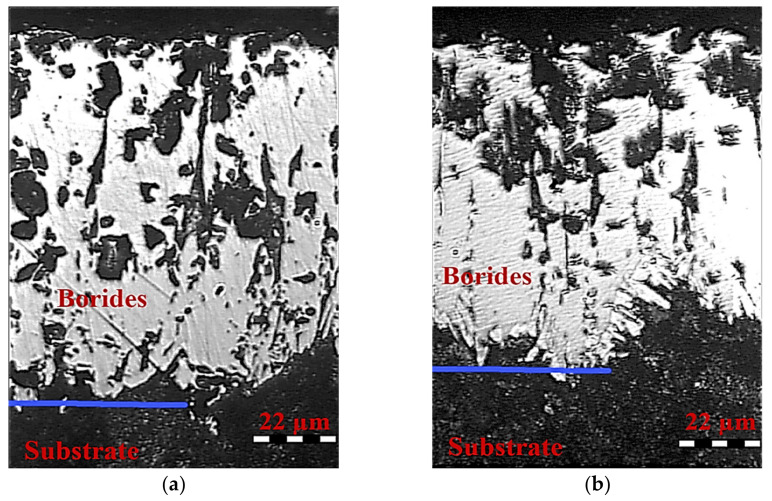

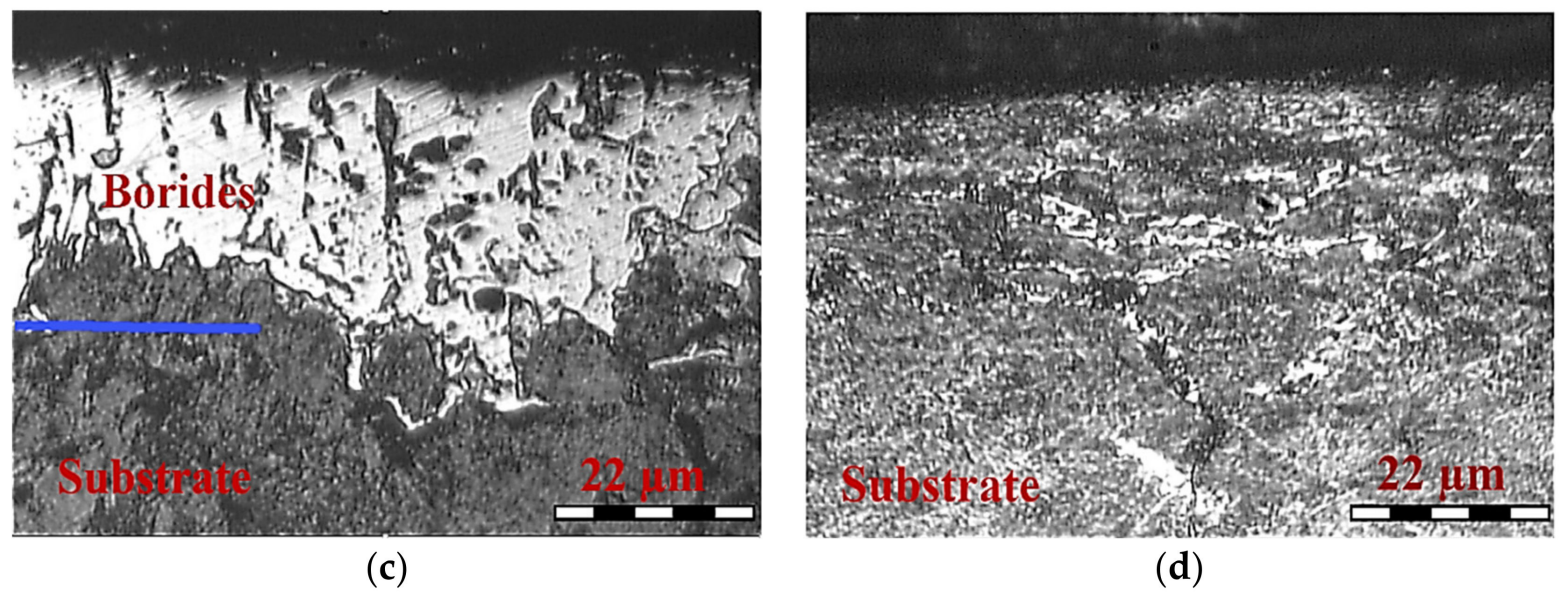
Microstructure of borided coating on steel C45 in the initial state (**a**) and after friction under specific pressure: (**b**) 3, (**c**) 7 and (**d**) 10 MPa.

The diffusion layer consists of borides. Boride layers have a high hardness of 1800–2000 HV (18,000–20,000 MPa), wear resistance (mostly abrasive), corrosion resistance and heat resistance (up to 764 °C). Boride aciculae grow into substrate material, thus forming a solid boride layer. Figure 3 shows the diffraction patterns of the borided layer after a wear test at various specific loads: a—3MPa; b—7 MPa; c—10 MPa. Fe_2_B boride was detected by all diffraction patterns. The borided zone has a typical double-phase structure that consists of FeB and Fe_2_B borides (light fields in Figure 4a).

With increasing boriding time, the quantity of FeB in the layer increased. Carbon present in steel considerably decreased FeB content in the layer. When the carbon content was higher, the size of the boride aciculae increased, and their ends become rounded. In the space between the FeB crystals, Fe_3_(C,B) boron alloyed cementite formed. After conventional etching, on micrographs it does not differ from iron borides. A transition area with a dissimilar structure may be observed. The heat-affected zone is a solid solution of boron in iron, and its thickness may be similar to the depth of boron penetration. In general, the thickness of the borided layer was 110–200 µm. The presence of a solid layer of borides and stress concentrators in the form of pores, cracks and grain boundaries resulted in rapid cracking in the case of high contact stresses. The influence of contact stresses extended to the transition zone, causing its plastic deformation. Plastic deformation of this zone, in turn, led to the crack propagation along the interphase boundary. As a result, disintegration of the borided occurred. Very hard debris penetrated the actual friction zone and caused high wear intensity. Figure 4 shows fragments of the borided coating after wear testing at 3, 7 and 10 MPa.

According to Figure 4, the thickness of the borided layer before wear test was 110 µm. The boride growth was perpendicular to the sample surface. The thickness of the boride layer after wear test at 7 MPa decreased in order of 3 (see Figure 4c). In many cases, the borided layer was practically absent. After wear testing at 10 MPa, the boride layer was absent over the whole surface of the specimen (Figure 4d). Traces of significant plastic deformation in the layers near the surface were not evident. As can be observed, the absence of the borided layer had an influence on the increase in wear intensity during testing at 10 MPa. Hence, the use of boriding in given working conditions is reasonable under specific pressure up to 7 MPa when wear loss is not significant (Figure 2).

**Figure 5:**
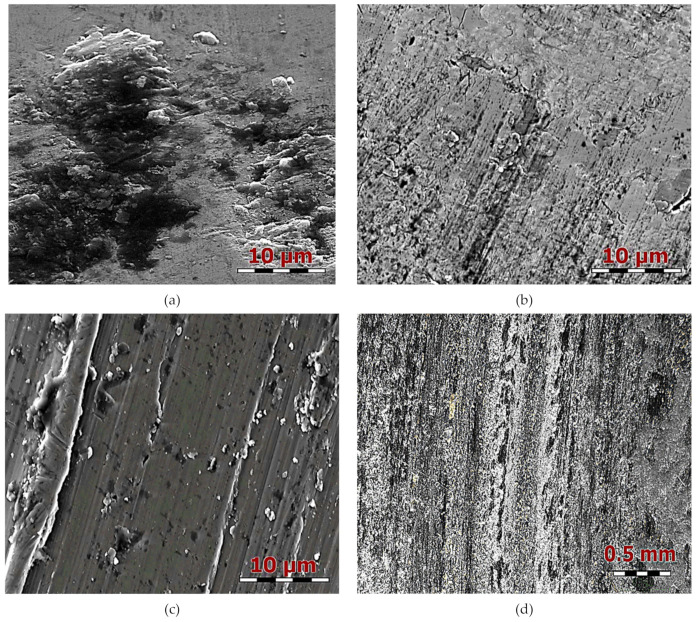
Microstructure of borided coating after wear test under specific pressure: (**a**) 3, (**b**) 7 and (**c**) 10 MPa; (**d**) counterpart under 3 MPa.

Identification of morphological traces of wear surface proves that in the borided coating (Figure 5a) under a specific pressure of 3 MPa, the oxidizing wear mechanism prevails. The surface had a specific leaf-film structure. It is clearly visible that the boundary layer consisted of several layers, which is the result of plastification of the material surface. Such a film appeared when the properties of the material were favorable (low yield strength, possible welding of wear products and their further spreading on the wear surface). The analysis of the wear surface after wear testing at 7 MPa showed that it had a lamellar structure (Figure 5b). Striations appeared as the result of the oriented movement of structural elements of the near-boundary layer. They were formed due to plastic being forced out of the material by hard particles present in the sublayer. The remaining regions of coating, as well as coating wear debris acted as abrasives, and the result of this action was visible on the surface. There were noticeable local dimples exceeding typical sizes of other structural components at the surface.

They were probably formed when the remaining borided layer, which has good adhesion with the substrate metal during displacement, met an obstacle on its way (e.g., plastic forced-out metal or other crumbled particle). During further interaction, the near-boundary layer tried to remove this inhomogeneity, and a particle was released from the surface and carried out of the friction zone, forming a tear-out.

While analyzing the wear surface under a pressure of 10 MPa (Figure 5c), it can be seen that it had groovings, or in some places sets of fine “scratches”. The formation of grooving structure was connected, first of all, with the intensive plastic deformation of the surfaces. This is usually formed as the result of friction between two plastic materials due to the localization of high specific pressures. A striated structure with fine “scratches” was formed because of intensive abrasion. Hard oxides, arising as a result of tribochemical reactions, may also appear as strong abrasives.

A counterpart surface analysis indicated that the surface had a striation-film structure with a partial delamination of the near-surface layer (Figure 5d). If oxidation wear prevails in given conditions, such film may be formed as a result of tribochemical reactions.

In order to evaluate which chemical elements available in coatings take part in the formation of the surface film, we conducted examinations of wear surface using secondary neutrals mass spectrometry (SIMS).

**Figure 6:**
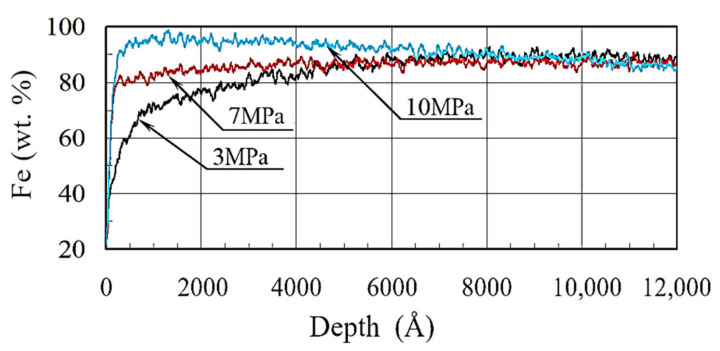
Iron distribution in borided layers under various specific pressures.

The influence of chemical elements B, O, C and Fe on the development of borided layers was considered. The main changes in the distribution of chemical elements occurred at the depth from 0 to 3000 Å. The change in the iron content in borided coatings is presented in Figure 6.

At the depth of ~ 50Å, a rapid decrease in iron concentration after wear testing at specific pressures of 3, 7 and 10 MPa was observed. However, a noticeable change in the concentration under different pressures proceeded differently. For example, at specific pressure of 3 MPa, a decrease in iron concentration from 90% down to 22.6% started at the depth of 6000 Å, and it continued to decrease towards the wear surface. At the specific pressure of 7 MPa, the decrease in concentration started at a depth of only 50 Å. The character of the iron concentration change that occurred under the specific pressure of 10 MPa was slightly different. At first, an increase in the iron concentration from 85% to 98% in the depth interval of 50–8000 Å and a rapid decrease in iron content down to 22.5% on the wear surface were observed. As can be seen, at different contact pressures, iron was differently involved in the oxide formation on the wear surface. Wear intensity had a significant influence on the character of iron concentration redistribution. For a more detailed analysis of the wear process, it is necessary to consider the influence of oxygen, carbon and boron on possible phase formation on the wear surface.

**Figure 7:**
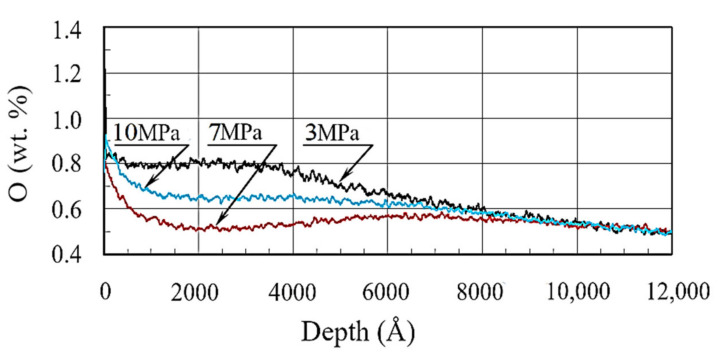
Oxygen distribution in borided layers under various specific pressures.

As can be seen from Figure 7, the maximum amount of oxygen on the surface was at a specific pressure of 3 MPa: 1.45% (Table 2). Oxygen concentration increased during testing with specific loads of 7 and 10 MPa. For the specific pressure of 7 MPa, the oxygen content increased from 0.55% at the depth of 800 Å to 0.8% at the surface.

**Figure 8:**
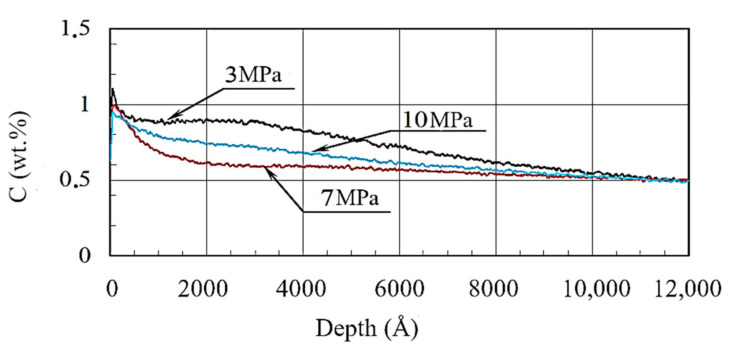
Carbon distribution in borided layers under various specific pressures.

Figure 8 indicates the increase in carbon content on the surface for all cases. Maximum carbon concentration was at a depth of ~100 Å for 3 MPa (1.04%), for 7 MPa it was 0.99%, and for 10 MPa it was 0.93%.

The character of carbon redistribution was similar to the curves of oxygen redistribution for all specific pressures. Certainly, the oxidation and increase in carbon content in surface layer are interrelated. In this case, the carbon layer was probably located underneath the oxide layer.

**Figure 9:**
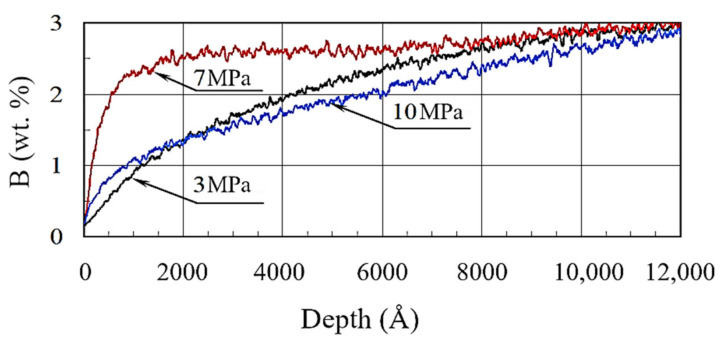
Distribution of boron content in borided layers under different specific pressures.

As boron is especially active in oxidation, the formation of non-stoichiometric B_2_O_3_ oxide on regions of actual friction contact is expected. This process justifies the boron redistribution with depth (Figure 9). Under specific pressures of 3 and 7 MPa, a smooth reduction in boron concentration from 2.9% to 0.2% across the coating depth was observed. However, under the specific pressure of 7 MPa, the decrease in boron concentration started at a depth of ~1000 Å, while for other cases, this decrease started at a depth of ~8000 Å.

Boron and iron oxides may form more complex oxide structures. Thus, it can be proved that under friction, the stoichiometric oxides based on B_2_O_3_-Fe_x_O_y_ or possible iron borides may form in the top layer of the borided diffusion zone.

The composition of the borided layer before and after wear test is shown in Table 2. Except basic B, C, O and Fe, many other chemical elements are present in the borided layer as admixtures that enter it during the boriding process. For solid-phase boriding, a powder mixture based on technical boron carbide and borax 84% B_4_C + 16% Na_2_B_4_O_7_ was used. Increased Na content was detected both inside the borided layer and on the wear surface. It was intook to the diffusion zone from the powder mixture used for thermochemical processing. Its content on the wear surface was greater than at a depth of 100 Å. Elements such as Cr, Si, Mn and Al, contained in the C45 steel, were also detected.

During tribological tests, the temperature was measured with a thermocouple (NiCr-NiAl). The thermocouple was placed 2 mm above the wear surface. After tribological tests with a load of 3 MPa, the maximum registered temperature was 38 °C. During friction at load of 7 MPa, the maximum temperature was 61 °C. During friction at load of 7 MPa, the registered temperature was much higher. The maximum temperature value for this case was 220 °C. However, it can be assumed that the actual temperature at the contact interface could reach much higher values.

Hence, after friction, the oxide film was formed under all test conditions. At the specific pressure of 3 MPa, a larger content of oxides was observed on the surface in comparison with 7 MPa. This confirms that under a specific pressure of 3 MPa the oxide wear mechanism prevails.

The surface film consists of stoichiometric and non-stoichiometric oxides based on Fe_x_O_y_ (Fe_3_O_4_ and Fe_2_O_3_) and B_2_O_3_, or possible borates. The oxide film is regularly damaged and mixed with fractured particles of borided layer. Under the specific pressure of 7 MPa, the abrasive wear dominates. Wear products escape the friction zone and deplete the surface of borides. The formation of such films under abrasive wear is accompanied by the decrease in wear intensity (Figure 2). Under specific pressures higher than 7 MPa, the wear process becomes more intensive, and areas of seizing and active mutual material transfer may be observed. The wear becomes mainly abrasive. Oxide films are regularly exposed to damage and under such loadings are not able to be recovered. As it can be seen in [35,36,44], similar C, B and Si atom segregation was observed during wear of Fe-Mn-C-B-Si-Ni-Cr eutectic alloys.

## 4. Conclusions

It was established that the borided coatings should be used at specific pressure of 7 MPa (0.74 g), as within this testing range the surface deterioration is insignificant and mass loss is moderate. The highest mass wear loss was for the borided surface after the wear test at contact pressures of 10 MPa: 2.26 g

It was revealed that the wear of a borided coating-steel C45 friction couple under the specific pressure of 3 MPa proceeds according to the oxidation mechanism, while under pressures of 7 and 10 MPa, the process has abrasive characteristics. The wear at higher pressures is beyond coating wear capabilities.

It was established that after testing under all specific pressures, oxide films are formed on the wear surface. On the actual contact areas, the surface film is formed and contains both stoichiometric and non-stoichiometric Fe_x_O_y_ and B_2_O_3_ oxides or possible borates. After wear testing under a specific pressure of 7 MPa, the boride layer deteriorates and wear particles act as abrasives. As a result, the oxide film formed is constantly damaged. Such a formation of films under abrasive wear is accompanied by a decrease in wear intensity. Possible B_2_O_3_-Fe_x_O_y_ and borates in corresponding friction conditions soften or pass into a liquid state and, additionally, assist in the decrease in the coating wear. These films prevent surface temperature exceeding B_2_O_3_ or possible iron borates melting points. The presence of carbon provides additional lubrication of the wear surface.

## Figures and Tables

**Table 1:** The average content of chemical elements in steel C45 (wt.%).

Material	Element (wt.%)						
	C	Mn	Si	Cr	Mo	Ni	Fe
C45	0.42–0.50	0.5–0.8	0.1–0.4	0.3	0.1	0.3	Balance

**Table 2:** Chemical composition (wt.%) of the borided coatings before and after wear test.

Chemical Element	Coating Nominal Composition (wt. %)	Coating Composition after Wear Test at Specific Pressure 3 MPa, (wt. %)		Coating Composition after Wear Test at Specific Pressure 3 MPa, (wt. %)		Coating Composition after Wear Test at Specific Pressure 3 MPa, (wt. %)	
		On Surface	On Depth of 100 Å	On Surface	On Depth of 100 Å	On Surface	On Depth of 100 Å
**Al**	2.7–4.4	7.6	10.9	3	3.2	6.6	3.76
**Si**	0.23–0.3	0.8	0.7	0.6	0.7	0.8	0.95
**Cr**	0.2–0.4	0.9	1	0.3	0.3	0.4	0.7
**Mn**	0.3–0.4	0.2	0.45	0.2	0.3	0.3	0.9
**Na**	2.3–5.1	7.2	6.4	51.8	14.1	18.1	8.6
**B**	2.9–3	0.2	0.2	0.2	0.6	0.2	0.4
**C**	0.5	0.92	1	0.7	0.99	0.6	0.9
**O**	0.5	1.45	0.8	0.8	0.8	0.8	0.9
**Fe**	Balance	22.6	42.1	19.9	61.7	21.5	62.5
